# Assessment of knowledge, attitude and practice towards rabies and associated factors among household heads in Mekelle city, Ethiopia

**DOI:** 10.1186/s12889-020-8145-7

**Published:** 2020-01-14

**Authors:** Weldegerima Gebremedhin Hagos, Kindie Fentahun Muchie, Goyitom Gebremdehn Gebru, Gebreamlak Gebremariam Mezgebe, Kebede Ambaye Reda, Berihun Assefa Dachew

**Affiliations:** 1 Directorate of Public Health Emergency Management, Tigray Regional Health Bureau, Mekelle, Ethiopia; 2 Department of Epidemiology and Biostatistics, University of Gonder, Gonder, Ethiopia; 3 Directorate of Laboratory Research, Diagnostics and Quality Assurance, Tigray Health Research Institute, Mekelle, Ethiopia; 4 Directorate of Health Reform and Good Governance, Tigray Regional Health Bureau, Mekelle, Ethiopia; 5 Directortate of Maternal, Neonatal and Child Health, Tigray Regional Health Bureau, Mekelle, Ethiopia

**Keywords:** Attitude, Knowledge, Practice, House hold, Rabies

## Abstract

**Background:**

Rabies has a worldwide distribution in continental regions of Africa, Asia and the Latin America. Globally, the case fatality rate is 100% once a clinical sign is developed. Poor public awareness towards rabies is one of the major obstacles in any prevention and control scheme of the diseases. The study aimed to assess knowledge, attitude and practice (KAP) about rabies and associated factors among household heads in Mekelle city, Northern Ethiopia, 2016.

**Methods:**

A community based cross-sectional study was conducted from October to November 2016 with a total of 633 study participants. Data were collected using a pretested structured questionnaire and entered to EPI-Info 3.5.4 and coded, cleaned and analyzed using SPSS version 20 software. Bi variable and multivariable analysis was done to identify factors associated with knowledge, attitude and practice about rabies. Variables having *p* < 0.05 was considered as statistically significant at 95%CI.

**Results:**

Of 633 study participants, 357 (56.4%) were females and 239 (37.8%) were 18–35 years old. Among the study participants, 56.1% (95%CI = 52.2, 59.9), 56.2% (95%CI = 52.4, 60.1) and 61.3% (95%CI = 57.5, 65.1) had good level of knowledge, attitude and practice on the prevention and control of rabies respectively. Being female (AOR = 1.50, 95%CI = 1.05, 2.13), dog owner (AOR = 1.68, 95%CI = 1.17, 2.41) and participants who had training on rabies (AOR = 2.22, 95%CI = 1.53, 3.21) were found to have good knowledge. Married participants (AOR = 2.19, 95%CI = 1.16, 4.16), participants who owned dog (AOR = 2.64, 95%CI = 1.80, 3.86) and those encountered dog bite (AOR = 2.24, 95%CI = 1.23, 4.10) were found to have positive attitude towards rabies. Similarly, dog ownership (AOR = 11.85, 95%CI = 7.16, 19.6) was found to be associated with good practice.

**Conclusion:**

This study showed that more than half of the respondents had good knowledge, attitude and practice about the prevention and control of rabies.

## Background

Rabies is one of the oldest viral disease caused by the species of rabies virus which belongs to the Mononegavirales order, Rhabdoviridae family and Lyssavirus genus [1]. It is a single-stranded, negative-sense lyssavirus (genotype 1) with a genome size of approximately 12 kb. Rabies causes incurable viral encephalitis and it is progressively fatal [2].

Human Rabies usually transmitted in saliva from a rabid animal bite or scratch. Following the bite the virus replicates in the muscle and travels up the central nervous system which causes infection of the brain (encephalitis). Although human rabies encephalitis is 100% fatal, it is also 100% preventable if post exposure prophylaxis (PEP) is taken timely and effectively by the exposed victims [3, 4]. Exposure to rabid animal can be eliminated at source through sustained mass vaccination of reservoir populations [3, 4].

Rabies affects any mammal mainly carnivores and insectivorous bats but dogs are the principal source of infection for humans. Globally, rabies causes more than 61,000 human deaths [5] and around 15 million dog bite victims receive post exposure prophylaxis every year [6]. Asia and Africa are the countries where more than 95% of the rabies mediated human deaths occur and 43% of the death occurred in Africa [7, 8]. Estimates of human mortality due to endemic canine rabies in Asia and Africa annually exceed 31,000 and 24,000 respectively [9].

In Ethiopia rabies is highly endemic, with an estimated 10,000 deaths annually [10]. In Ethiopia, particularly in Tigray region, accurate quantitative information on animal and human rabies is limited [11]. In addition, little is known about the awareness of the people regarding rabies, which makes the effective implementation of prevention and control measures challenging [12].

In public health knowledge, attitude and practice (KAP) studies have been widely used based on the principle that increasing knowledge will result in changing attitude and practice to minimize disease burden [13]. In Tigray region, like the other areas of Ethiopia, there are a number of home and street dogs. Many households owned dogs mainly for a security purpose particularly in the rural areas but also in the urban areas of the region. Despite this, regular vaccination and follow-up to the dogs are not being given [14]. In addition to this, there is no study conducted on KAP of rabies prevention and control in Mekelle city. Therefore, the aim of this study was to fill the gap on the availability of data regarding status of KAP towards rabies among household heads in Mekelle city, Northern Ethiopia.

## Methods

### Study area

The study was conducted in Mekelle city, Tigray Region, Northern Ethiopia. Mekelle city is the capital city of Tigray Region and it is found about 783 km far from Addis Ababa. According to the projected census of 2007 Ethiopian Fiscal Year, the city had a total population of 340,589. The city is divided into seven administrative sub cities namely: Hawelti, Hadnet, Ayder, Semen, Kedamay weyane, Adihaki, and Quiha.

### Study design and study period

A community based cross-sectional study was conducted from October 3, 2016 to November 29, 2016.

### Sample size determination

The sample size was calculated using single population proportion formula. Assuming the proportion of knowledge level 83%, attitude level 52.3%, and practice level 67% [8] with 5% margin of error, 95% confidence level, and 1.5 design effect, the calculated sample size for knowledge, attitude and practice were 217, 383 and 340 respectively.

Therefore, the larger sample size among the knowledge, attitude and practice is taken as appropriate which is 383. Then, the sample size 383 is multiplied by 1.5 design effect (385*1.5 = 575) and 10% none response rate was added to 575. Finally, the final sample size was determined to be 633.

### Sampling procedure

**Fig. 1:**
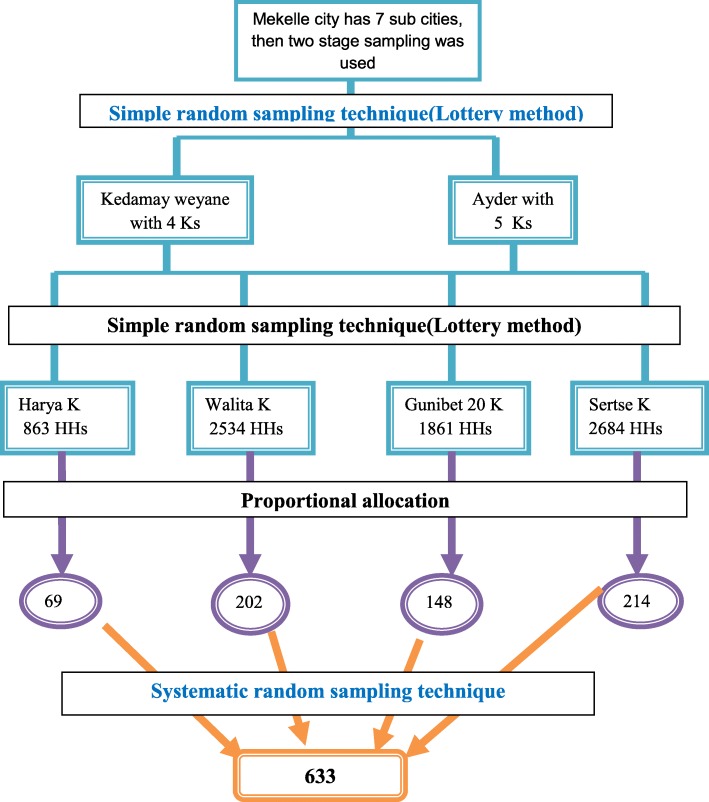
Sampling procedure for assessing knowledge, attitude and practice about rabies and associated factors among household heads in Mekelle city, Northern Ethiopia, 2016

Mekelle city has seven sub cities namely Hawelti, Hadnet, Ayder, Semen, Kedamay weyane, Adihaki, and Quiha. Among the seven sub cities, two were selected using the simple random sampling technique (lottery method), namely Kedamay weyane (with four kebelles) and Ayder (with five kebelles). Two kebelles for each sub city (total four kebelles) were selected for the study by lottery method (Fig. 1).

The selected kebelles were *Harya* and *Walita* from Kedamay weyane sub city and *Gunibet 20* and *Seritse* from Ayder sub city. Therefore, the total sample size was proportionally allocated to the four kebelles based on 6 months living in the kebelles (Fig. 1). Therefore, the study participants were household heads. List of the households or each kebelles were found from the respective sub city administrative. The first household was selected randomly between 1 and 13 using lottery method, then the first household to be included in the sample was the fifth household, then every 13th households was selected. In case, a selected household’s head was not found at home, a second visit was made by appointment.

### Data collection procedure

A pretested structured questionnaire (Additional file 1) was used. Originally, the questionnaire was written in English and translated into local language (Tigrigna) and retranslated into English to test for any differences or inconsistencies in the meaning of words and concepts. Data were collected by trained nurses using face to face interview technique.

Twenty three questions (eight knowledge, seven attitude and eight practice) were asked for each participant regarding cause, sources and mode of transmissions, attitude, practice and prevention and control measures about rabies. The questions to assess knowledge and practice were in type of a response of either “Yes” or “No” for each question. Whereas for attitude questions, the likert scale method with a five points scale (strongly agree, agree, no opinion, disagree, strongly disagree) responses were used to allow the study participants to express how much they agree or disagree with a particular statement. Responses with strongly agree and agree had got one mark, while responses with disagree and strongly disagree had got zero mark. For a participant with a response of “no opinion”, we exclude the question from the summing of the overall score for the participant.

The participant’s response was counted and graded separately for the questions of knowledge, attitude, and practice. This score was then pooled and the mean score was calculated to determine respondent’s knowledge, attitude and practice. Accordingly, mean scores for knowledge, attitude, and practice were calculated as 5.5, 4.6 and 1.9 respectively.

Respondents who score greater than or equal to the mean value for knowledge, attitude, and practice were grouped to have good knowledge, positive attitude and good practice respectively. Whereas, respondents who score less than the mean value for knowledge, attitude, and practice were grouped to have poor knowledge, negative attitude and poor practice respectively.

### Data quality control

A structured questionnaire was pretested on 5% sample size outside of the study area, in Enderta District, for consistent understanding of the survey. Close supervision was undertaken during data collection. Questionnaire was checked for completeness and consistency before data entry by the principal investigator.

### Data management and analysis

Data were entered and cleaned in EPI-info version 3.5.4 and exported in to SPSS version 20 for analysis. Descriptive analyses were used to see the frequency distribution of the socio-demographic and economic factors. Bivariate logistic regression analysis was used to identify the association between dependent and independent variables. Variables with *p*-value of 0.20 and less in the bivariate logistic regression were taken to multivariate logistic model for multivariable analysis. In the multivariate analysis, variables with p-value less than 0.05 were considered as statistically significant.

### Ethical consideration

Ethical clearance was obtained from the Ethical Review Board of Institute of Public Health, College of Medicine and Health Science, University of Gondar with a reference number of IPH/2284/09/2016. Official permission was obtained from Tigray Regional Health Bureau, Mekelle Zone, Kedamay weyane and Ayder sub cities. All study participants were adult and verbal consent was obtained from each study participant to confirm willingness for participation after explaining the objective of the study. Confidentiality of the information was maintained throughout by excluding names and keeping their privacy during the interview, by interviewing them alone. Participants had the right to withdraw at any time from the interview.

### Operational definition

**Good knowledge**: respondents who scored points at mean and above for the knowledge questions prepared were referred to be having good knowledge otherwise not.

**Positive attitude**: respondents who scored points at mean and above for the attitude questions were referred to be having a positive attitude otherwise not.

**Good practice**: respondents who scored points at mean and above for the practice questions were referred to be having a good practice otherwise not.

**Kebelle**: is the smallest administrative unit of Ethiopia.

## Results

### Socio-demographic and economic factors

A total of 633 household heads were interviewed in this research, which yields a response rate of 100%. More than half 357 (56.6%) of the interviewed participants were females. Regarding age group, the age of the respondents range from 19 to 80 with a mean age of 42.3 ± 13.8 and the majority 271 (42.8%) of participants age were between 36 and 55 years old (Additional file 2: Table S1).

### Access to health information and environmental factors towards rabies

The study participants reported that, they get information regarding rabies from different formal and informal sources (Table 1).

**Table 1:** Access to health information and environmental factors towards rabies of study participants in Mekelle city, northern Ethiopia

Variables	Frequency	%
Dog ownership		
Yes	256	40.4
No	377	59.6
Family exposure to dog bite		
Yes	89	14.1
No	544	85.9
Has get training/awareness about rabies		
Yes	208	32.9
No	425	67.1
Source of information about rabies		
Formal (News paper, TV/radio)	107	17
Informal	267	42.2
Mixed Source	187	29.5
Governmental rabies vaccine campaigns	54	8.5
No information about rabies	18	2.8
Main reservoir/source of rabies		
Dog	624	98.6
Cat	147	23.2
Other domestic animals	160	24.5

### Knowledge of participants towards rabies

This study revealed that 555(87.8%) of respondents had heard information about rabies (Additional file 2: Table S1). About 470 (74.2%) of respondents said that rabies affects all warm blooded animals including human, 423 (66.8%) of participants said that dog rabies vaccine could be obtained from authorized governmental institutions (Additional file 3: Table S2).

### Attitude towards rabies

This study revealed that 525 (82.9%) of respondents said that stray dogs are dangerous and 360 (56.9%) were willing to register their pets. About 536 (84.7%) of respondents were annoyed with stray dogs. Majority 557 (88%) were said rabies prevented by health education and 315 (49.8%) respondents believed holly water cure rabies (Additional file 4: Table S3).

### Practice of participants towards rabies

This study showed that, 383 (60.5%) of participants have contact with dogs and cats and 351 (60.5%) participants have hand washing habit after touching dogs and cats(Additional file 5: Table S4). Among 89 respondents who had ever been bitten by a dog or who had a family member ever bitten by a dogs, only 69 (77.5%) went to health institution after bite as followed by 11 (12.3%) went to holly water (Additional file 5: Table S4).

### Knowledge, attitude and practice related to rabies

**Fig. 2:**
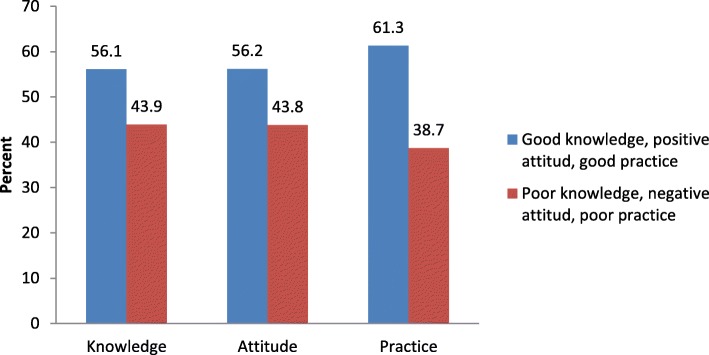
Knowledge, attitude and practice towards rabies among household heads in Mekelle city, northern Ethiopia

According to the study 56.1% (95%CI = 52.2, 59.9), 56.2% (95%CI = 52.4, 60.1) and 61.3% (95%CI = 57.5, 65.1) of participants had good knowledge, positive attitude and good practice towards rabies respectively, as shown in Fig. 2.

### Factors associated with knowledge

Variables including sex, occupation, dog ownership, training, monthly income, educational status and exposed family to dog bite with *p*-value less than 0.2 in bivariate analysis were entered in to multivariable binary logistic regression analysis model (Additional file 6: Table S5).

The multivariable analysis result of this study declared that sex, occupation, dog ownership, training and monthly income had statistically significant association with knowledge about rabies at 5% level of significance.

### Factors associated with attitude

Variables including marital status, educational status, household size, dog ownership, exposure of family to dog bite, monthly income and knowledge with *p*-value less than 0.2 in bivariate analysis were entered in to multivariable binary logistic regression analysis model (Additional file 7: Table S6).

The multivariable analysis result of this study revealed that marital status, educational status, household size, dog ownership and family exposure to dog bite had statistically significant association with attitude about rabies at 5% level of significance (Additional file 7: Table S6).

### Factors associated with practice

Variables including marital status, educational status, age, household size, knowledge, attitude, dog ownership and family exposure to dog bite with p-value less than 0.2 in bivariate analysis were entered in to multivariable binary logistic regression analysis model (Additional file 8: Table S7).

The multivariable analysis result of this study revealed that dog ownership and exposure family to dog bite had statistically significant association with practice about rabies at 5% level of significance (Additional file 8: Table S7).

According to this study, educational status, age, household size, knowledge and attitude were not significantly associated with practice about rabies in the multivariable analysis (Additional file 8: Table S7).

## Discussion

This study revealed that, total mean score for practice, attitude and knowledge was 61.3, 56.2 and 56.1% respectively.

Of the 633 respondents, 88.2% had heard about rabies before exposure. This suggests that victims are aware of the presence of rabies in their area. Majority (88.9%) of the victims had heard about rabies from informal sources (family, friends and neighbors), which is similar with study conducted in Tanzania [13].

Among the study participants, 79% of household heads had vaccinated their dogs. This finding is similar with study conducted in Indonesia (74%) [15] and SriLanka (76%) [16]. However, this result is higher than study conducted in Kenya (35%) [17], Jimma (4.8%) [18], Gondar 42% [19] and Dessie (35.8%) towns in Ethiopia [20]. This may be attributed to a number of factors that include availability of animal vaccines, the study time and good information sharing in this study area. Household heads with good knowledge about rabies were 56.1% (95%CI = 52.2, 59.9), which was lower than studies conducted in SriLanka (89.6%) [16], Indonesia (82.6%) [15], Guatemala (82%) [21], Tanzania (96%) [13], Addis Ababa (83%) [8], Bahir-Dar (60.1%) [10], Gondar (90.8%) [19] and Debretabor (65.1%) [9]. The possible reasons for this difference could be due to low health promotion particularly regarding rabies in this study area.

Among the household heads in this study, 56.2% (95%CI = 52.4, 60.1) had positive attitude about rabies, which was lower than study conducted in Indonesia (96%) [15]. This difference probably might be explained by the lack of health education about rabies in the study site. Moreover, attitude of the current finding is greater than the study conducted in Bahir-Dar (42.8%) [8], Addis Ababa (52.3%) [8] and Debretabor (40.6%) [9]. This may be due to time difference which could bring a difference on awareness of study participants.

This study revealed that female household heads were 1.5 times more likely (AOR = 1.5, 95%CI = 1.05, 2.13) to have good knowledge towards rabies as compared to male household heads. This finding was similar with study conducted in Addis Ababa [8] and Jimma town [18]. This could be due to the reason that females get awareness about rabies from house to house by urban health extension workers, women development army, giving health education in health institution and better chance of acquiring correct information about rabies.

On the other hand, governmental employee household heads were almost two times more likely (AOR = 1.96, 95%CI = 1.03, 3.73) to have good knowledge towards rabies as compared to those unemployed household heads. This may be due to the reason that employees are more educated than unemployed. This study’s finding was lower than a study conducted in Tanzania [13] and Nigeria [22]. This may be due to the difference in frequency and way of information dissemination in the community.

Household heads who had dogs in their household were 68% more likely (AOR = 1.68, 95%CI = 1.17, 2.41) to have good knowledge toward rabies as compared with counterpart study participants. This finding was in line with a study conducted in Tanzania [13]. The possible justification for this finding could be those who have dogs got good information about rabies in the time of vaccination, on how to care dogs and prevent rabies exposure.

According to this study having a secondary school educational level (9–12 grade) and higher education (diploma and above) were 58 and 73% less likely (AOR = 0.42, 95%CI = 0.18, 0.97) to have positive attitude towards rabies as compared to those who do not read and write. This finding was lower than the study conducted in Tanzania [13], Namibia [23] and Nigeria [22]. This might be due to variation on level of awareness towards rabies.

Those who have dogs were 11.85 times more likely (AOR = 11.85, 95%CI = 7.16, 19.6) to have good practice toward rabies prevention as compared to those who had not dogs. This study is in line with the study conducted in Tanzania [13]. Those communities have got good awareness about rabies in the time of vaccination, care of dogs, and this may help them to have good practice about rabies prevention and control.

In this study, we did not address all the questions related with KAP. In spite of the study limitation, the study is still significant in showing the level of knowledge, attitude and practice in Mekelle city. The finding of this study elicits further awareness creation program through different mechanism for effective prevention of rabies in the community.

## Conclusions

This study showed that more than half of the study participants had good knowledge, attitude and practice about rabies. This indicates a significant amount of the study participants lack awareness on rabies. Therefore, further awareness creation activities and multi-sectoral collaborations to prevent rabies are needed in the region.

Furthermore, this significant lack of awareness calls for further study on the health burden of rabies in the region.

## Supplementary information

**Additional file 1:.** A questionnaire for the Assessment of Knowledge, Attitude and Practice about rabies and associated factors among household heads, Mekelle, Tigray, Ethiopia, 2016.

**Additional file 2: Table S1.** Socio-demographic and economic factors of study participants.

**Additional file 3: Table S2.** Computed knowledge variables of study participants to ward rabies in Mekelle city, northern Ethiopia.

**Additional file 4: Table S3.** Computed attitudes of study participants toward rabies in Mekelle city, northern Ethiopia.

**Additional file 5: Table S4.** Computed practice variables of study participants toward rabies in Mekelle city, northern Ethiopia.

**Additional file 6: Table S5.** Factors associated with knowledge towards rabies among household heads in Mekelle city, northern Ethiopia.

**Additional file 7: Table S6.** Factors associated with attitude towards rabies among study participants in Mekelle city, northern Ethiopia.

**Additional file 8: Table S7.** Factors associated with practice towards rabies among study participants in Mekelle city, northern Ethiopia.

## Data Availability

The authors ensure the availability of data and material of this research work and are ready to provide by contacting the corresponding author on a reasonable request.

## Abbreviations

AOR: Adjusted Odds Ratio
CI: Confidence Interval
COR: Crude Odds Ratio
EPHI: Ethiopian Public Health Institute
KAP: Knowledge, Attitude and Practice
PEP: Post Exposure Prophylaxis
