# Supplementary material for: Assessment of knowledge, attitude and practice towards rabies and associated factors among household heads in Mekelle city, Ethiopia
Source: BMC Public Health. 2020 Jan 14;20:57. doi: 10.1186/s12889-020-8145-7 (PMC6961227; doi:10.1186/s12889-020-8145-7)
Supplement: Supplementary file 1 — Additional file 1:. A questionnaire for the Assessment of Knowledge, Attitude and Practice about rabies and associated factors among household heads, Mekelle, Tigray, Ethiopia, 2016. [file 12889_2020_8145_MOESM1_ESM.docx]

## Additional file 1: A questionnaire for the Assessment of Knowledge, Attitude and Practice about rabies and associated factors among household heads, Mekelle, Tigray, Ethiopia, 2016

A questionnaire used to assess knowledge, attitudes and practices about rabies and associated factors among household heads in Mekelle city (Kedemay weyane and Ayder sub cities) Northern Ethiopia, 2016.

**I. Socio-demography, economic characteristics and environment and access to health information of participants**

| **No.** | **Question** | | **Response** | | **Skip** | |
| --- | --- | --- | --- | --- | --- | --- |
| 101 | Sex | | 1. Male  2. Female | |  | |
| 102 | Age in years | | ---------------- | |  | |
| 103 | Household size | | ---------------- | |  | |
| 104 | Ethnicity | | 1. Tigre  2. Amahara  3. Oromo  4. Afar  5. Others, specify........................... | |  | |
| 105 | Religion | | 1. Orthodox  2. Muslim  3. Protestant  4. Catholic  5. Others, specify........................... | |  | |
| 106 | Marital status | | 1. Married  2. Unmarried  3. Divorced  4. Widowed  5. Separated | |  | |
| 107 | Educational status | | 1. Not read and write  2. Read and write  3. Primary  4. Secondary  5. Higher education (Diploma & above) | |  | |
| 108 | Occupation | | 1. Government employee  2. Private employee  3. Merchant  4. Housewife  5. Farmer  6. Student  7. Unemployed  8. Others specify........................... | |  | |
| 109 | Monthly income in birr | | ........................ | |  | |
| 110 | Dog ownership | | 1. Yes  2. No | |  | |
| 111 | Family exposure to dog bite | | 1. Yes  2. No | |  | |
| **II. Knowledge towards rabies related to cause, mode of transmissions and host range, clinical signs, prevention & control, treatment and fatal nature of rabies** | | | | | | |
| **No.** | **Question** | | | **Response** | | **Skip** |
| 201 | Have you ever heard about rabies disease? | | | 1. Yes  2. No  3. I don’t know | | If no skip to Q203 |
| 202 | Source of information for rabies | | | 1. Mass media (News paper, TV/radio)  2. Non Mass media (Traditionally information change)  3. Mixed Source (Both)  4. Government rabies vaccination campaigns  5. Others, specify........................... | |  |
| 203 | Does/ can rabies be prevented by vaccination? | | | 1. Yes  2. No  3. I don’t know | |  |
| 204 | Can rabies be treated by post exposure prophylaxis? | | | 1. Yes  2. No  3. I don’t know | |  |
| 205 | Do you know about the almost 100% fatal nature of rabies once the clinical signs develop/ developed? | | | 1. Yes  2. No | |  |
| 206 | Do you know that you and your family are at risk of getting rabies if the dog is not vaccinated? | | | 1. Yes  2. No | |  |
| 207 | Have you ever get training about rabies? | | | 1. Yes  2. No | |  |
| 208 | Do you know that rabies can affect all warm blooded animals including human beings? | | | 1. Yes  2. No | |  |
| 209 | What is the cause of rabies? | | | 1. Psychological problem  2. Bacteria  3. Virus  4. Shortage of food and water  5. I don't know  6. Others specify.................. | |  |
| 210 | Do you know that dog rabies vaccine could be obtained from authorized government offices? | | | 1. Yes  2. No | |  |
| 211 | What is the mode of transmission of rabies from rabid animal to human?  you can circle more than one answer | | | 1. Bite by rabid dog only  2. Scratching only  3. Contact with saliva  4. Bite and saliva contact with open wound  5. I don’t know  6. Others specify.................. | |  |
| 212 | Which is the main reservoir/source of rabies?  you can circle more than one answer | | | 1. Dog  2. Cat  3. Bat  4. Other domestic animal  5. I don’t know  6. Others specify.................. | |  |
| 213 | What are the clinical sign and symptoms of rabies in humans?  you can circle more than one answer | | | 1. Stops eating and drinking  2. Biting and sudden change in behavior  3. Paralysis  4. Madness  5. Salivation  6. Hydrophobia  7. I don’t know  8. Others specify.................. | |  |
| 214 | At which stage of anti-rabies vaccine is effective after a suspected animal bite? | | | 1. Immediately  2. Later  3. At any time  4. I don’t know  5. Others specify.................. | |  |
| 215 | Do you Know that the head of suspected animal must be submitted to laboratory for confirmation? | | | 1.Yes  2. No | |  |
| 216 | **Is it good to wash dog bite wounds with soap and water?** | | | 1.Yes  2. No | |  |
| 217 | Which groups of people are at higher risk of rabies?  you can circle more than one answer | | | 1. Children  2.Young  3. Adult  4. Male  5. Female  6. I don’t know | |  |
| **III. Attitude of community towards rabies** | | | | | | |
| 301 | | Stray dogs are dangerous | | 1. Strongly agree 2. Agree 3. No opinion 4. Disagree 5. Strongly disagree | |  |
| 302 | | Rabies is a problem in your kebelle for your community | | 1. 1. Strongly agree 2. Agree 3. No opinion 4. Disagree 5. Strongly disagree | |  |
| 303 | | Do you believe holly water can treat rabies disease? | | 1. 1. Strongly agree 2. Agree 3. No opinion 4. Disagree 5. Strongly disagree | |  |
| 304 | | Rabies can be effectively prevented by euthanizing (killing) stray dogs | | 1. Strongly agree 2. Agree 3. No opinion 4. Disagree 5. Strongly disagree | |  |
| 305 | | Rabies can be prevented by educating people about the disease | | 1. Strongly agree 2. Agree 3. No opinion 4. Disagree 5. Strongly disagree | |  |
| 306 | | Willing to register pets | | 1. Strongly agree 2. Agree 3. No opinion 4. Disagree 5. Strongly disagree | |  |
| 307 | | Annoyed with stray dogs | | 1. Strongly agree 2. Agree 3. No opinion 4. Disagree 5. Strongly disagree | |  |
| **IV. Practice of community towards rabies** | | | | | | |
| 401 | | Do any of your family members touch dog or cat(s)? | | 1.Yes  2. No | |  |
| 402 | | Do any of your family members wash their hands after touching the dog(s), or cat(s)? | | 1.Yes  2. No | |  |
| 403 | | Have you ever been bitten by a dog? | | 1.Yes  2. No | | If no skip to Q405 |
| 404 | | If you get bitten by a dog where do you go first? | | 1. Stay at home  2. To health institution  3. To holly water  4. To traditional Healers  5. Others specify.................. | |  |
| 405 | | Which measures do you take to control stray dogs? | | 1. Aware the owner  2. Killing  3. Animal birth control  4. Ting  5. Others specify.................. | |  |
| 407 | | Would you inform authorities if bitten by dog? | | 1. Yes  2. No | |  |
| 408 | | Did you vaccinate your dog? | | 1.Yes  2. No | | If no skip to Q410 |
| 409 | | Can you able to show the dog vaccination certificate? | | 1. Yes  2. No | |  |
| 410 | | Where do you care your dog? | | 1. Housed in cages  2. Tied outside the house  3. Free living inside the house  4. Free to roam around  5. Others specify................ | |  |
