# Supplementary material for: Assessment of knowledge, attitude and practice towards rabies and associated factors among household heads in Mekelle city, Ethiopia
Source: BMC Public Health. 2020 Jan 14;20:57. doi: 10.1186/s12889-020-8145-7 (PMC6961227; doi:10.1186/s12889-020-8145-7)
Supplement: Supplementary file 2 — Additional file 2: Table S1. Socio-demographic and economic factors of study participants. [file 12889_2020_8145_MOESM2_ESM.docx]

Additional file 2: Table 1: Socio-demographic and economic factors of study participants

| **Variables** | **Frequency** | **%** |
| --- | --- | --- |
| Age | | |
| 18-35 | 239 | 37.8 |
| 36-55 | 271 | 42.8 |
| 56-75 | 116 | 18.3 |
| >75 | 7 | 1.1 |
| **Sex** | | |
| Male | 276 | 43.6 |
| Female | 357 | 56.4 |
| **Ethnicity** | | |
| Tigre | 576 | 91 |
| Amhara | 57 | 9 |
| **Marital status** | | |
| Married | 365 | 57.7 |
| Unmarried | 116 | 18.3 |
| Divorced | 92 | 14.5 |
| Widowed | 60 | 9.5 |
| **Educational status** | | |
| Do not read and write | 45 | 7.1 |
| Read and write | 89 | 14.1 |
| Primary school complete | 188 | 29.7 |
| Secondary school complete | 162 | 25.6 |
| Higher education | 149 | 23.5 |
| **Religion** | | |
| Orthodox-Christian | 540 | 85.3 |
| Muslim | 78 | 12.3 |
| Catholic | 15 | 2.4 |
| **Occupation** | | |
| Government employee | 140 | 22.1 |
| Private employee | 130 | 20.5 |
| Merchant | 133 | 21 |
| House wife | 88 | 13.9 |
| Farmer | 26 | 4.1 |
| Student | 39 | 6.2 |
| Unemployed | 77 | 12.2 |
| **Household size** | | |
| **1-3** | 327 | 51.7 |
| **4-6** | 264 | 41.7 |
| **>6** | 42 | 6.6 |
| **Average monthly income in birr** | | |
| <1000 | 165 | 26 |
| 1000-2000 | 187 | 29.5 |
| >2000 | 281 | 44.5 |
