# Supplementary material for: Assessment of knowledge, attitude and practice towards rabies and associated factors among household heads in Mekelle city, Ethiopia
Source: BMC Public Health. 2020 Jan 14;20:57. doi: 10.1186/s12889-020-8145-7 (PMC6961227; doi:10.1186/s12889-020-8145-7)
Supplement: Supplementary file 3 — Additional file 3: Table S2. Computed knowledge variables of study participants to ward rabies in Mekelle city, northern Ethiopia. [file 12889_2020_8145_MOESM3_ESM.docx]

Additional file 3: Table S2: Computed knowledge variables of study participants to ward rabies in Mekelle city, northern Ethiopia

| **Variables** | **Frequency** | **%** |
| --- | --- | --- |
| **Heard about rabies disease** | | |
| Yes | 555 | 87.7 |
| No | 78 | 12.3 |
| **Rabies affect all warm blooded animals including human** | | |
| Yes | 470 | 74.2 |
| No | 163 | 25.8 |
| **Dog rabies vaccine could be obtained from governmental institutions** | | |
| Yes | 423 | 66.8 |
| No | 210 | 33.2 |
| **Rabies prevented by vaccination** | | |
| Yes | 442 | 69.8 |
| No | 178 | 28.1 |
| I don't know | 13 | 2.1 |
| **Rabies treated by Post Exposure Prophylaxis** | | |
| Yes | 428 | 67.6 |
| No | 184 | 29.1 |
| I don't know | 21 | 3.3 |
| **Almost 100% fatal nature of rabies** | | |
| Yes | 289 | 45.7 |
| No | 344 | 54.3 |
| **Wash dog bite wounds with soap and water** | | |
| Yes | 382 | 60.3 |
| No | 251 | 39.7 |
| **Your family are at risk of getting rabies if the dog is not vaccinated** | | |
| Yes | 507 | 80.1 |
| No | 126 | 19.9 |
