# Supplementary material for: Assessment of knowledge, attitude and practice towards rabies and associated factors among household heads in Mekelle city, Ethiopia
Source: BMC Public Health. 2020 Jan 14;20:57. doi: 10.1186/s12889-020-8145-7 (PMC6961227; doi:10.1186/s12889-020-8145-7)
Supplement: Supplementary file 4 — Additional file 4: Table S3. Computed attitudes of study participants toward rabies in Mekelle city, northern Ethiopia. [file 12889_2020_8145_MOESM4_ESM.docx]

Additional file 4: Table S3: Computed attitudes of study participants toward rabies in Mekelle city, northern Ethiopia

| **Variables** | Frequency | % |
| --- | --- | --- |
| **Stray dogs are dangerous** | | |
| Agree* | 525 | 82.9 |
| Disagree** | 108 | 17.1 |
| **Rabies is a problem in your kebelle** | | |
| Agree* | 244 | 38.5 |
| Disagree** | 389 | 61.5 |
| **Holly water can treat rabies disease** | | |
| Agree* | 315 | 49.8 |
| Disagree** | 318 | 50.2 |
| **Rabies can be effectively prevented by killing stray dogs** | | |
| Agree* | 394 | 62.2 |
| Disagree** | 239 | 37.8 |
| **Rabies prevented by educating people** | | |
| Agree* | 557 | 88 |
| Disagree** | 76 | 12 |
| **Willing to register pets** | | |
| Agree* | 360 | 56.9 |
| Disagree** | 273 | 43.1 |
| **Annoyed with stray dogs** | | |
| Agree* | 536 | 84.7 |
| Disagree** | 97 | 15.3 |

**Note:** * Participants response with strongly agree and agree are merged as “Agree”

**Participants response with disagree and strongly disagree are merged as “Disagree”
