# Supplementary material for: Assessment of knowledge, attitude and practice towards rabies and associated factors among household heads in Mekelle city, Ethiopia
Source: BMC Public Health. 2020 Jan 14;20:57. doi: 10.1186/s12889-020-8145-7 (PMC6961227; doi:10.1186/s12889-020-8145-7)
Supplement: Supplementary file 5 — Additional file 5: Table S4. Computed practice variables of study participants toward rabies in Mekelle city, northern Ethiopia. [file 12889_2020_8145_MOESM5_ESM.docx]

Additional file 5: Table 5: Computed practice variables of study participants toward rabies in Mekelle city, northern Ethiopia

| **Variables** | **Frequency** | **%** |
| --- | --- | --- |
| **Your family members touch dogs and cats** | | |
| Yes | 383 | 60.5 |
| No | 250 | 39.5 |
| **Your family members wash their hands after touching the dog, cat** | | |
| Yes | 351 | 55.5 |
| No | 282 | 44.5 |
| **Would you inform authorities if you were bitten by dog** | | |
| Yes | 82 | 92.2 |
| No | 7 | 7.8 |
| **Vaccinated your dog** | | |
| Yes | 195 | 79.3 |
| No | 51 | 20.7 |
| **Do you have dog vaccination certificate of your dog** | | |
| Yes | 151 | 77.4 |
| No | 44 | 22.6 |
| **Ever been bitten by a dog** | | |
| Yes | 89 | 14.1 |
| No | 544 | 85.9 |
| **When you get bit where did you go first** | | |
| Stay at home | 0 | 0 |
| To health institution | 69 | 77.5 |
| To holly water | 11 | 12.4 |
| To traditional Healers | 9 | 10.1 |
| **Measure taken to control stray dogs** | | |
| Aware the owner | 45 | 18.3 |
| Killing | 81 | 32.9 |
| Animal birth control | 14 | 5.7 |
| Incarceration | 106 | 43.1 |
| **Care your dog on** | | |
| Housed in cages | 179 | 72.8 |
| Tied outside the house | 38 | 15.4 |
| Free living inside the house | 21 | 8.5 |
| Free to roam around | 8 | 3.3 |
