# Supplementary material for: Assessment of knowledge, attitude and practice towards rabies and associated factors among household heads in Mekelle city, Ethiopia
Source: BMC Public Health. 2020 Jan 14;20:57. doi: 10.1186/s12889-020-8145-7 (PMC6961227; doi:10.1186/s12889-020-8145-7)
Supplement: Supplementary file 6 — Additional file 6: Table S5. Factors associated with knowledge towards rabies among household heads in Mekelle city, northern Ethiopia. [file 12889_2020_8145_MOESM6_ESM.docx]

Additional file 6: Table 6: Factors associated with knowledge towards rabies among household heads in Mekelle city, northern Ethiopia

| **Variables** | | **Knowledge** | | **COR 95% CI** | **AOR 95% CI** | **P-value** |
| --- | --- | --- | --- | --- | --- | --- |
|  |  | **Good n (%)** | **Poor n (%)** |  |  |  |
| **Sex** | Male | 140(50.7%) | 136(49.3%) | 1 | 1 |  |
|  | Female | 215(60.2%) | 142(39.8%) | 1.47(1.07, 2.02) | 1.50(1.05, 2.13)* | 0.03 |
| **Educational status** | Not read & write | 22(48.9%) | 23(51.1%) | 1 | 1 |  |
|  | Read & write | 41(46.1%) | 48(53.9%) | 0.89(0.44, 1.83) | 1.09(0.50, 2.41) | 0.83 |
|  | Primary | 103(54.8%) | 85(45.2%) | 1.27(0.66, 2.43) | 1.57(0.75, 3.27) | 0.23 |
|  | Secondary | 99(61.1%) | 63(38.9%) | 1.64(0.85, 3.19) | 1.98(0.93, 4.22) | 0.08 |
|  | Higher education | 90(60.4%) | 59(39.6%) | 1.60(0.82, 3.12) | 1.77(0.81, 3.86) | 0.15 |
| **Occupation** | Government employee | 86(61.4%) | 54(38.6%) | 1.81(1.03, 3.18) | 1.96(1.03, 3.73)* | 0.04 |
|  | Private employee | 76(58.5%) | 54(41.5%) | 1.60(0.91, 2.83) | 1.39(0.75, 2.60) | 0.29 |
|  | Merchant | 73(54.9%) | 60(45.1%) | 1.39(0.79, 2.43) | 1.38(0.74, 2.56) | 0.31 |
|  | House wife | 50(56.8%) | 38(43.2%) | 1.50(0.81, 2.77) | 1.37(0.71, 2.64) | 0.35 |
|  | Farmer | 10(38.5%) | 16(61.5%) | 0.71(0.29, 1.76) | 0.77(0.29, 2.00) | 0.56 |
|  | Student | 24(61.5%) | 15(38.5%) | 1.82(0.83, 4.00) | 1.36(0.58, 3.19) | 0.48 |
|  | Unemployed | 36(46.8%) | 41(53.2%) | 1 | 1 |  |
| **Average monthly income birr** | <1000 | 99(60%) | 66(40%) | 1 | 1 |  |
|  | 1001-2000 | 94(50.3%) | 93(49.7%) | 0.67(0.44, 1.03) | 0.61(0.38, 0.96)* | 0.03 |
|  | >2000 | 162(57.7) | 119(42.3%) | 0.91(0.61, 1.34) | 0.64(0.41, 1.02) | 0.09 |
| **Dog ownership** | Yes | 164(64.1%) | 92(35.9%) | 1.74(1.25, 2.40) | 1.68(1.17, 2.41)* | 0.01 |
|  | No | 191(50.7%) | 186(49.3) | 1 | 1 |  |
| **Exposure fam. to dog bite** | Yes | 61(68.5%) | 28(31.5%) | 1.85(1.15, 2.99) | 1.56(0.92, 2.65) | 0.10 |
|  | No | 294(54%) | 250(46%) | 1 | 1 |  |
| **Training/awareness rabies** | Yes | 145(69.7%) | 63(30.3%) | 2.36(1.66, 3.35) | 2.22(1.53, 3.21)* | 0.00 |
|  | No | 210(49.4) | 215(50.6%) | 1 | 1 |  |

**Note**: Superscript indicates statistical significance *p-value < 0.05
