# Supplementary material for: Assessment of knowledge, attitude and practice towards rabies and associated factors among household heads in Mekelle city, Ethiopia
Source: BMC Public Health. 2020 Jan 14;20:57. doi: 10.1186/s12889-020-8145-7 (PMC6961227; doi:10.1186/s12889-020-8145-7)
Supplement: Supplementary file 7 — Additional file 7: Table S6. Factors associated with attitude towards rabies among study participants in Mekelle city, northern Ethiopia. [file 12889_2020_8145_MOESM7_ESM.docx]

Additional file 7: Table 7: Factors associated with attitude towards rabies among study participants in Mekelle city, northern Ethiopia

| **Variables** | | **Attitude** | | **COR 95% CI** | **AOR 95% CI** | **P-value** |
| --- | --- | --- | --- | --- | --- | --- |
|  |  | **Positive** | **Negative** |  |  |  |
| **Marital status** | Married | 215(58.9%) | 150(41.1%) | 1.43(0.83, 2.48) | 2.19(1.16, 4.16)* | 0.03 |
|  | Unmarried | 53(45.7%) | 63(54.3%) | 0.84(0.45, 1.57) | 1.46(0.70, 3.02) | 0.39 |
|  | Divorced | 58(63%) | 34(37%) | 1.71(0.88, 3.30) | 2.70(1.27, 5.75)* | 0.02 |
|  | Windowed | 30(50%) | 30(50%) | 1 | 1 |  |
| **Educational status** | Not read & write | 33(73.3%) | 12(26.7%) | 1 | 1 |  |
|  | Read & write | 57(64%) | 32(36%) | 0.65(0.29, 1.43) | 0.90(0.37, 2.19) | 0.76 |
|  | Primary | 123(65.4%) | 65(34.5%) | 0.69(0.33, 1.42) | 0.83(0.36, 1.90) | 0.57 |
|  | Secondary | 85(52.5%) | 77(47.5%) | 0.40(0.19, 0.83) | 0.42(0.18, 0.97)* | 0.03 |
|  | Higher education | 58(38.9%) | 91(61.1%) | 0.23(0.11, 0.49) | 0.27(0.12, 0.65)* | 0.00 |
| **Average monthly income birr** | <_1000 | 100(60.6%) | 65(39.4%) | 1 | 1 |  |
|  | 1001-2000 | 114(61%) | 73(39%) | 1.02(0.66, 1.56) | 1.19(0.73, 1.93) | 0.50 |
|  | >2000 | 142(50.5%) | 139(49.5%) | 0.66(0.45, 0.98) | 0.82(0.52, 1.33) | 0.36 |
| **House hold size** | 1-3 | 191(58.4%) | 136(41.6%) | 1.87(0.98, 3.59) | 3.22(1.51, 6.90)* | 0.00 |
|  | 4-6 | 147(55.7%) | 117(44.3%) | 1.68(0.87, 3.22) | 2.70(1.26, 5.78)* | 0.01 |
|  | >6 | 18(42.9%) | 24(57.1%) | 1 | 1 |  |
| **Dog ownership** | Yes | 182(71.1%) | 74(28.9%) | 2.87(2.05, 4.02) | 2.64(1.80, 3.86)* | 0.00 |
|  | No | 174(46.2%) | 203(53.8%) | 1 | 1 |  |
| **Exposure fam. to dog bite** | Yes | 69(77.5%) | 20(22.5%) | 3.09(1.83, 5.23) | 2.24(1.23, 4.10)* | 0.01 |
|  | No | 287(52.8%) | 257(47.2%) | 1 | 1 |  |
| **Knowledge** | Good | 141(50.7%) | 137(49.3%) | 1.49(1.09, 2.05) | 1.42(0.99, 2.03) | 0.05 |
|  | Poor | 215(60.6%) | 140(39.4%) | 1 | 1 |  |

**Note**: Superscript indicates statistical significance *p-value < 0.05
