# Supplementary material for: Assessment of knowledge, attitude and practice towards rabies and associated factors among household heads in Mekelle city, Ethiopia
Source: BMC Public Health. 2020 Jan 14;20:57. doi: 10.1186/s12889-020-8145-7 (PMC6961227; doi:10.1186/s12889-020-8145-7)
Supplement: Supplementary file 8 — Additional file 8: Table S7. Factors associated with practice towards rabies among study participants in Mekelle city, northern Ethiopia. [file 12889_2020_8145_MOESM8_ESM.docx]

Additional file 8: Table 8: Factors associated with practice towards rabies among study participants in Mekelle city, northern Ethiopia

| **Variables** | | **Practice** | | **COR 95% CI** | **AOR 95% CI** | **P-value** |
| --- | --- | --- | --- | --- | --- | --- |
|  |  | **good** | **poor** |  |  |  |
| **Educational status** | Not read & write | 33(73.3%) | 12(26.7%) | 1 | 1 |  |
|  | Read & write | 52(58.4%) | 37(41.6%) | 0.51(0.23, 1.12) | 0.69(0.27, 1.76) | 0.43 |
|  | Primary | 113(60.1%) | 75(39.9%) | 0.55(0.27, 1.13) | 0.45(0.19, 1.15) | 0.09 |
|  | Secondary | 107(66%) | 55(34%) | 0.71(0.34, 1.48) | 0.70(0.28, 1.76) | 0.45 |
|  | Higher education | 83(55.7%) | 66(44.3%) | 0.46(0.22, 0.95) | 0.54(0.22, 1.38) | 0.20 |
| **Age** | 18-35 | 149(62.3%) | 90(37.7%) | 0.28(0.03, 2.33) | 0.61(0.05, 6.86) | 0.67 |
|  | 36-55 | 169(62.4%) | 102(37.6%) | 0.28(0.30, 2.33) | 0.45(0.04, 4.97) | 0.52 |
|  | 56-75 | 64(55.2%) | 52(44.8%) | 0.21(0.02, 1.76) | 0.29(0.26, 3.27) | 0.32 |
|  | >75 | 6(85.7%) | 1(14.3%) | 1 | 1 |  |
| **House hold size** | 1-3 | 193(59%) | 134(41%) | 0.45(0.21, 0.95) | 0.55(0.22, 1.39) | 0.21 |
|  | 4-6 | 163(61.7%) | 101(38.3%) | 0.50(0.24, 1.07) | 0.60(0.24, 1.52) | 0.28 |
|  | >6 | 32(76.2%) | 10(23.8%) | 1 | 1 |  |
| **Dog ownership** | Yes | 233(91%) | 23(9%) | 14.5(9.02, 23.3) | 11.8(7.16, 19.6)* | 0.00 |
|  | No | 155(41.1%) | 222(58.9%) | 1 | 1 |  |
| **Exposure fam. to dog bite** | Yes | 83(93.3%) | 6(6.7%) | 10.8(4.65, 25.3) | 5.25(2.09, 13.2)* | 0.00 |
|  | No | 305(56.1%) | 239(43.9%) | 1 | 1 |  |
| **knowledge** | Good | 152(54.7%) | 126(45.3%) | 1.64(1.19, 2.27) | 1.20(0.81, 1.79) | 0.36 |
|  | Poor | 236(66.5%) | 119(33.5%) | 1 | 1 |  |
| **Attitude** | Positive | 142(51.3%) | 135(48.7%) | 2.13(1.54, 2.95) | 1.35(0.91, 2.01) | 0.14 |
|  | Negative | 246(69.1%) | 110(30.9%) | 1 | 1 |  |

**Note**: Superscript indicates statistical significance *p-value < 0.05
